# Supplementary material for: Accelerating Chemical Potential Calculations with Minimal Normalizing Flows
Source: J Chem Theory Comput. 2026 Sep 25;22(19):9976–91. doi: 10.1021/acs.jctc.6c01266 (PMC13671182; doi:10.1021/acs.jctc.6c01266)
Supplement: Supplementary file 1 [file ct6c01266_si_001.pdf]

Accelerating Chemical Potential Calculations with Minimal Normalizing Flows : Supplementary Information

Philippe B. Baron and Athanassios Z. Panagiotopoulos*

Department of Chemical and Biological Engineering, Princeton University, Princeton, NJ, 08540

E-mail: azp@princeton.edu

S1 Simulation Details for all Test Cases

Here we specify all of the details of the MD simulations in LAMMPS used for all of the test cases.[1] For the pure Lennard-Jones (LJ) simulations, we conduct simulations in the NVT ensemble, using a Langevin thermostat with a damping constant of 0.1 ps. We use a time-step of 1 fs. LJ interactions are truncated and shifted at a cutoff radius of $r_c = L/2$, where $L = (N\sigma^3/\rho^*)^{1/3}$. We use a 2 Å neighbor list skin distance. The potential energy of our inserted particle is evaluated using the LAMMPS "soft-core" LJ potential given by —

$$u_{ij}^{\text{LJ, soft}}(r_{ij}, n, C) = 4\epsilon_{ij}\lambda^n \left[\frac{1}{\left(C(1-\lambda)^2 + \left(\frac{r_{ij}}{\sigma_{ij}}\right)^6 \right)^2} - \frac{1}{C(1-\lambda)^2 + \left(\frac{r_{ij}}{\sigma_{ij}}\right)^6} \right] \tag{S1}$$

where we set $n = 1$ and $C = 1$. Simulations are equilibrated for 500 ps before a production run of 20 ns. Ground truth free energies are computed using 10 equally spaced windows corresponding to $\lambda = \{0.0, 0.1, \ldots, 1.0\}$.

Table S1: ϵ and σ parameters for the binary LJ mixture system studied by Espinosa *et al.*[2]

**Interaction**	ϵ/k_B **(K)**	σ **(Å)**
A-A	114.128	3.405
A-B	74.406	3.798
B-B	61.691	3.405

For the binary LJ test case, we simulate under NPT conditions at a pressure of $P = 1$ atm and temperature $T = 50$ K. LJ interactions are truncated and shifted at a cutoff radius of $r_c = 8.5125$ Å, and force field (FF) parameters for this system are given in Table S1. We use a Langevin thermostat with a damping constant of 0.2 ps and a Nosé-Hoover barostat with a damping constant of 2 ps. We use a time-step of 2 fs. The inserted particle again has "soft-core" LJ interactions as given by Eq. S1, with $n = 1$ and $C = 1$. Simulations are equilibrated for 4 ns before a production run of 40 ns. Ground truth free energies are computed using 10 equally spaced windows corresponding to $\lambda = \{0.0, 0.1, \ldots, 1.0\}$.

Table S2: Force-field parameters for the binary SPC/E water + Joung-Cheatham ion model.[3,4] Water geometry is held rigid with $r_{\text{OH}} = 1.0$ Å and $\theta_{\text{HOH}} = 109.47°$. Cross interactions are determined using the Lorentz-Berthelot mixing rules — $\epsilon_{ij} = \sqrt{\epsilon_i \epsilon_j}$ and $\sigma_{ij} = 0.5(\sigma_i + \sigma_j)$.

**Atom**	ϵ **(kcal/mol)**	σ **(Å)**	q **(e)**
Na^+	0.3526	2.1595	1.0000
Cl^-	0.0128	4.8305	−1.0000
O	0.1554	3.1660	−0.8476
H	0.0000	–	0.4238

For the electrolyte test case, we also simulate under NPT conditions at a pressure of $P = 1$ atm and temperature $T = 300$ K. LJ interactions are truncated at a cutoff radius of $r_c = 8.0$ Å, and FF parameters are provided in Table S2. We use a Langevin thermostat with a damping constant of 0.2 ps and a Nosé-Hoover barostat with a damping constant of 2 ps. We use a time-step of 2 fs. The SHAKE algorithm was used to constrain SPC/E

water geometry. The inserted particle again has "soft-core" LJ interactions as given by Eq. S1, with $n = 2$ and $C = 0.5$. Simulations are equilibrated for 10 ns before a production run of 60 ns. Long-range Coulombic interactions are calculated using the PPPM method with an error tolerance of 1.0×10^{-6}. Ground truth charging free energies are computed using 20 equally spaced windows corresponding to $\phi = \{0.0, 0.05, \ldots, 1.0\}$. Ground truth FF perturbation free energies are computed using 10 equally spaced windows corresponding to $\lambda = \{0.0, 0.1, \ldots, 1.0\}$, applied to the interpolating potential $U(\lambda) = (1-\lambda)U(\boldsymbol{\theta}_0) + \lambda U(\boldsymbol{\theta}_1)$, where $\boldsymbol{\theta}_i$ is the ion FF parameter set at state i.

S2 Correlation Function Decomposition of the Kullback-Leibler Divergence for a Pure Fluid

We begin by assuming we are in a pure system of N atoms under NVT conditions (with number density $\rho = N/V$) interacting via a LJ potential ($u_{\mathrm{LJ}}(r)$ given as the first term of Eq. 27 in the main text), where the interactions of one of the atoms (indexed 1) is controlled by a coupling parameter $\lambda \in [0, 1]$. The system potential energy then reads —

$$U_i(\mathbf{r}_1, \ldots, \mathbf{r}_N) = \lambda_i \sum_{k=2}^{N} u_{\mathrm{LJ}}(r_{1k}) + \sum_{k=2}^{N} \sum_{j>k} u_{\mathrm{LJ}}(r_{kj}) \tag{S2}$$

where $\lambda_0 = 0.0$ and $\lambda_1 = 1.0$. We evaluate the ensemble averages that appear in Eq. 14 of the main text, starting with the first term which corresponds to the forward Kullback-Leibler Divergence (KLD) under the influence of a 1D radial mapping relative to the inserted solute

(as given in Eq. 17 of the main text) —

$$
\begin{aligned}
&\langle \beta U_1(\phi(x)) - \ln|\det \mathcal{J}(x)|\rangle_0 = \int \left[\beta U_1(\phi(x)) - \ln|\det \mathcal{J}(x)|\right] \frac{e^{-\beta U_0(x)}}{Z_0} dx \\
&= \int \left[\sum_{k=2}^{N}\left[\beta u(\psi(r_{1k})) - \ln\left[\frac{\psi(r_{1k})^2\psi'(r_{1k})}{r_{1k}^2}\right]\right] + \beta \sum_{k=2}^{N}\sum_{j>k} u(\mathit{\Phi}(\mathbf{r}_1, \mathbf{r}_k, \mathbf{r}_j))\right] \frac{e^{-\beta U_0(x)}}{Z_0} dx \\
&= \underbrace{\int \sum_{k=2}^{N}\left[\beta u(\psi(r_{1k})) - \ln\left[\frac{\psi(r_{1k})^2\psi'(r_{1k})}{r_{1k}^2}\right]\right] \frac{e^{-\beta U_0(x)}}{Z_0} dx}_{\text{solute-solvent}} \\
&+ \underbrace{\int \left[\beta \sum_{k=2}^{N}\sum_{j>k} u(\mathit{\Phi}(\mathbf{r}_1, \mathbf{r}_k, \mathbf{r}_j))\right] \frac{e^{-\beta U_0(x)}}{Z_0} dx}_{\text{solvent-solvent}}
\end{aligned}
\tag{S3}
$$

where we neglect the $-\langle U_0(x)\rangle_0 - \Delta F$ terms that are independent of our mapping, we implement the Jacobian expression from Eq. 18 of the main text, $u(r)$ refers to $u_{\text{LJ}}(r)$ for brevity, and $\mathit{\Phi}(\mathbf{r}_1, \mathbf{r}_k, \mathbf{r}_j)$ is a function that captures the effect of the radial mapping away from the solute on the distance between two solvent particles —

$$
\mathit{\Phi}(\mathbf{r}_1, \mathbf{r}_k, \mathbf{r}_j) = \sqrt{\psi\left(r_{1j}\right)^2 + \psi\left(r_{1k}\right)^2 - 2\psi\left(r_{1j}\right)\psi\left(r_{1k}\right)\cos\theta} \tag{S4}
$$

where θ is the angle between the vectors $\mathbf{r}_{1j}$ and $\mathbf{r}_{1k}$. We first look closer at the solute-solvent term, defining a "mapped" solute-solvent potential given by, $\tilde{u}(r) = \beta u(\psi(r)) - \ln\left[\frac{\psi(r)^2\psi'(r)}{r^2}\right]$, which allows us to re-write the solute-solvent mapped energy —

$$
\begin{aligned}
\int \sum_{k=2}^{N} \tilde{u}(r_{1k}) \frac{e^{-\beta U_0(x)}}{Z_0} dx &= (N-1) \int \tilde{u}(r_{12}) \frac{e^{-\beta U_0(x)}}{Z_0} dx \\
&= (N-1) \int\int \tilde{u}(r_{12}) \frac{\int \cdots \int e^{-\beta U_0(x)} d\mathbf{r}_3 \cdots d\mathbf{r}_N}{Z_0} d\mathbf{r}_1 d\mathbf{r}_2 \\
&= (N-1) \int\int \tilde{u}(r_{12}) \frac{\rho^2 g_0^{(2)}(\mathbf{r}_1, \mathbf{r}_2)}{N(N-1)} d\mathbf{r}_1 d\mathbf{r}_2 \\
&= 4\pi\rho \int \left[\beta u(\psi(r)) - \ln\left[\frac{\psi(r)^2\psi'(r)}{r^2}\right]\right] g_0^{(2)}(r) r^2 dr
\end{aligned}
\tag{S5}
$$

Now we can re-write the solvent-solvent term —

$$
\begin{aligned}
&\int \left[\sum_{k=2}^{N}\sum_{j>k} u(\Phi(\mathbf{r}_1,\mathbf{r}_k,\mathbf{r}_j))\right]\frac{e^{-\beta U_0(x)}}{Z_0}dx = \binom{N-1}{2}\int u(\Phi(\mathbf{r}_1,\mathbf{r}_2,\mathbf{r}_3))\frac{e^{-\beta U_0(x)}}{Z_0}dx \\
&= \binom{N-1}{2}\int\int\int u(\Phi(\mathbf{r}_1,\mathbf{r}_2,\mathbf{r}_3))\frac{\int\cdots\int e^{-\beta U_0(x)}d\mathbf{r}_4\cdots d\mathbf{r}_N}{Z_0}d\mathbf{r}_1 d\mathbf{r}_2 d\mathbf{r}_3 \\
&= \binom{N-1}{2}\int\int\int u(\Phi(\mathbf{r}_1,\mathbf{r}_2,\mathbf{r}_3))\frac{\rho^3 g_0^{(3)}(\mathbf{r}_1,\mathbf{r}_2,\mathbf{r}_3)}{N(N-1)(N-2)}d\mathbf{r}_1 d\mathbf{r}_2 d\mathbf{r}_3 \\
&= \frac{\rho^2}{2V}\int\int\int u(\Phi(\mathbf{r}_1,\mathbf{r}_2,\mathbf{r}_3))g_0^{(3)}(\mathbf{r}_1,\mathbf{r}_2,\mathbf{r}_3)d\mathbf{r}_1 d\mathbf{r}_2 d\mathbf{r}_3 \\
&= \frac{\rho^2}{2}\int\int u(\Phi(\mathbf{r}_{12},\mathbf{r}_{13}))g_0^{(3)}(\mathbf{r}_{12},\mathbf{r}_{13})d\mathbf{r}_{12}d\mathbf{r}_{13} \\
&= \frac{\rho^2}{2}\int\int\int u(\Phi(r_{12},r_{13},\theta))g_0^{(3)}(r_{12},r_{13},\theta)4\pi r_{12}^2 r_{13}^2\sin(\theta)d\phi d\theta dr_{12}dr_{13} \\
&= 4\pi^2\rho^2\int\int\int u(\Phi(r_{12},r_{13},\theta))g_0^{(3)}(r_{12},r_{13},\theta)r_{12}^2 r_{13}^2\sin(\theta)d\theta dr_{12}dr_{13}
\end{aligned}
\tag{S6}
$$

where in both of these steps we have employed the standard definition of $g_i^{(n)}$ as the n-body correlation function in ensemble i between the "inserted" particle and $n-1$ solvent particles.[5] This gives our final expression for the ensemble average of interest —

$$
\begin{aligned}
\langle\beta U_1(\phi(x)) - \ln|\det\,\mathcal{J}(x)|\rangle_0 = 4\pi\rho\int\left[\beta u(\psi(r)) - \ln\left[\frac{\psi(r)^2\psi'(r)}{r^2}\right]\right]g_0^{(2)}(r)r^2dr \\
+4\pi^2\rho^2\beta\int\int\int u(\Phi(r,r',\theta))g_0^{(3)}(r,r',\theta)r^2r'^2\sin(\theta)d\theta dr dr'
\end{aligned}
\tag{S7}
$$

This is the expression given in Eq. 19 of the main text. Now looking at the second ensemble average term in Eq. 14 of the main text (corresponding to the KLD for the reverse direction), we analogously have —

$$
\begin{aligned}
&\left\langle \beta U_0(\phi^{-1}(y)) - \ln\left|\det\, \mathcal{J}^{-1}(y)\right|\right\rangle_1 = \int \left[\beta U_0(\phi^{-1}(y)) - \ln\left|\det\, \mathcal{J}^{-1}(y)\right|\right] \frac{e^{-\beta U_1(y)}}{Z_1} dy \\
&= \int \left[\beta \sum_{k=2}^{N} \sum_{j>k} u(\Phi^{-1}(\mathbf{r}_1, \mathbf{r}_k, \mathbf{r}_j)) - \sum_{k=2}^{N} \ln\left[\frac{\psi^{-1}(r_{1k})^2 (\psi^{-1})'(r_{1k})}{r_{1k}^2}\right]\right] \frac{e^{-\beta U_1(y)}}{Z_1} dy \\
&= \underbrace{-\int \sum_{k=2}^{N} \ln\left[\frac{\psi^{-1}(r_{1k})^2 (\psi^{-1})'(r_{1k})}{r_{1k}^2}\right] \frac{e^{-\beta U_1(y)}}{Z_1} dy}_{\text{solute-solvent}} + \underbrace{\int \left[\beta \sum_{k=2}^{N} \sum_{j>k} u(\Phi^{-1}(\mathbf{r}_1, \mathbf{r}_k, \mathbf{r}_j))\right] \frac{e^{-\beta U_1(y)}}{Z_1} dy}_{\text{solvent-solvent}}
\end{aligned}
\tag{S8}
$$

where similarly to the forward direction, we have neglected the terms $-\langle U_1(y)\rangle_1 + \Delta F$ as they are independent of the mapping parameters. Now we can proceed in the same way as for the forward case and express the ensemble average in terms of two and three-body correlations —

$$
\begin{aligned}
\left\langle \beta U_0(\phi^{-1}(y)) - \ln\left|\det\, \mathcal{J}^{-1}(y)\right|\right\rangle_1 &= -4\pi\rho \int \ln\left[\frac{\psi^{-1}(r)^2 (\psi^{-1})'(r)}{r^2}\right] g_1^{(2)}(r) r^2 dr \\
&+ 4\pi^2\rho^2\beta \int\int\int u(\Phi^{-1}(r, r', \theta)) g_1^{(3)}(r, r', \theta) r^2 r'^2 \sin(\theta) d\theta dr dr'
\end{aligned}
\tag{S9}
$$

S3 Comparisons of the Harmonic Distance (HD) and the Bhattacharyya Distance (BD)

S3.1 Dependence on Free Energy Estimate

Assume we have our harmonic distance ($\mathbb{D}_{\text{HD}}$) as defined in Eq. 21 of the main text, and let us assume that we are inputting a biased free energy estimate $\Delta\hat{F} = \Delta F + \epsilon$ into this loss at the beginning of training. Thus, our loss is (considering the forward direction without loss of generality) —

$$
\mathbb{D}_{\text{HD}}(\theta, \epsilon) = -\ln \left\langle \frac{2}{1 + \exp\left[-\beta\left(\Delta F + \epsilon - \tilde{W}^{(F)}\right)\right]} \right\rangle_0
\tag{S10}
$$

where for simplicity we consider dependence only on a single scalar "minimal" normalizing flow (MNF) parameter θ. Thus, we can say that the optimal MNF parameter set $\theta^*(\epsilon)$ is the one that satisfies the stationarity condition —

$$\left\langle \frac{\partial}{\partial\theta}\left(\frac{1}{1+\exp\left[-\beta\left(\Delta F+\epsilon-\tilde{W}^{(F)}\right)\right]}\right)\right\rangle_0 = 0 \tag{S11}$$

where we neglect multiplicative factors that do not affect the minimum. For simplicity, we refer to samples of the dissipation term as
$\omega(x,\theta,\Delta\hat{F}) = \beta\left(\Delta F+\epsilon-\tilde{W}^{(F)}(x,\theta)\right)$. We can also define the sigmoid function $\sigma(x) = 1/(1+e^{-x})$ and it is a known identity $\sigma'(x) = \sigma(x)(1-\sigma(x))$, which gives us —

$$F(\theta,\epsilon) = \left\langle \sigma\left(\omega\right)\left(1-\sigma\left(\omega\right)\right)\frac{\partial\tilde{W}^{(F)}}{\partial\theta}\right\rangle_0 = 0 \tag{S12}$$

Here we note that if we have a fully expressive mapping, the global minimum will be a perfect map from one state to the other. By the Crooks fluctuation theorem,[6] we can see that this means that all mapped works will be equal to the free energy difference, which gives $\omega = \beta\epsilon$ for all configurations x. Thus, this makes our optimality condition —

$$\left\langle \sigma\left(\beta\epsilon\right)\left(1-\sigma\left(\beta\epsilon\right)\right)\frac{\partial\tilde{W}^{(F)}}{\partial\theta}\right\rangle_0 = 0 \Rightarrow \left\langle \frac{\partial\tilde{W}^{(F)}}{\partial\theta}\right\rangle_0 = 0 \tag{S13}$$

now independent of the bias, and this condition is simply a saturation of the bound in Eq. 15 in the main text. While the bias does not affect the minimum in the limit of a perfect mapping, we can see that for a non-expressive map like a MNF, ϵ will affect the solution of Eq. S12. This is because whenever the mapped work distribution is not a delta function centered at ΔF (the mapping is not ideal), the term $\sigma\left(\omega\right)\left(1-\sigma\left(\omega\right)\right)$ cannot be factored out of the ensemble average in Eq. S12. Particularly, the ensemble average in Eq. S12 will now preferentially weight derivatives of works in the region near $W \approx \Delta F+\epsilon$ rather than $W \approx \Delta F$.

If desired by the reader, one can obtain more concrete expressions for the shift by expressing our stationarity condition as $F\left(\theta^*(\epsilon), \epsilon\right) = 0$, thus by differentiating both sides with respect to ϵ we have that —

$$\frac{d\theta^*}{d\epsilon} = -\left(\frac{\partial F}{\partial \theta}\right)^{-1} \frac{\partial F}{\partial \epsilon} \tag{S14}$$

where we assume that we have a well defined minimum ($\partial F/\partial\theta \neq 0$). This quantifies the dependence of the optimal parameter set on the bias ϵ.

S3.2 Gradient Behavior

We can write the harmonic distance ($\mathbb{D}_{\text{HD}}$, Eq. 21 in the main text) and the Bhattacharyya distance ($\mathbb{D}_{\text{BD}}$, Eq. 22 in the main text) as follows (considering the forward direction, without loss of generality) —

$$\mathbb{D}_{\text{BD}} = -\ln\left\langle e^{\frac{\mathcal{W}}{2}}\right\rangle_0 \tag{S15}$$

$$\mathbb{D}_{\text{HD}} = -\ln\left\langle \frac{2}{1+e^{-\mathcal{W}}}\right\rangle_0 = -\ln\left\langle e^{\frac{\mathcal{W}}{2}}\operatorname{sech}\left(\frac{\mathcal{W}}{2}\right)\right\rangle_0 \tag{S16}$$

Now, we know that the quantity $\mathcal{W}$ is a function of our mapping parameters θ. We would like to compare the magnitudes of the derivatives of each distance in the low overlap regime where all samples of $\mathcal{W}_i$ are far from 0. Thus, let us evaluate the derivatives —

$$\frac{\partial \mathbb{D}_{\text{BD}}}{\partial \theta} = -\frac{\left\langle \frac{1}{2}e^{\frac{\mathcal{W}}{2}}\frac{\partial \mathcal{W}}{\partial \theta}\right\rangle_0}{\left\langle e^{\frac{\mathcal{W}}{2}}\right\rangle_0} \tag{S17}$$

$$
\begin{aligned}
\frac{\partial \mathbb{D}_{\mathrm{HD}}}{\partial \theta} &= -\frac{\left\langle \frac{1}{2}e^{\frac{\mathcal{W}}{2}}\frac{\partial \mathcal{W}}{\partial \theta}\operatorname{sech}\left(\frac{\mathcal{W}}{2}\right) - \frac{1}{2}e^{\frac{\mathcal{W}}{2}}\frac{\partial \mathcal{W}}{\partial \theta}\operatorname{sech}\left(\frac{\mathcal{W}}{2}\right)\tanh\left(\frac{\mathcal{W}}{2}\right)\right\rangle_0}{\left\langle e^{\frac{\mathcal{W}}{2}}\operatorname{sech}\left(\frac{\mathcal{W}}{2}\right)\right\rangle_0} \\
&= -\frac{\left\langle \frac{1}{2}e^{\frac{\mathcal{W}}{2}}\frac{\partial \mathcal{W}}{\partial \theta}\operatorname{sech}\left(\frac{\mathcal{W}}{2}\right)\left[1-\tanh\left(\frac{\mathcal{W}}{2}\right)\right]\right\rangle_0}{\left\langle e^{\frac{\mathcal{W}}{2}}\operatorname{sech}\left(\frac{\mathcal{W}}{2}\right)\right\rangle_0}
\end{aligned}
\tag{S18}
$$

For the forward direction, we have that $\left\langle \tilde{W}^{(F)} \right\rangle_0 \geq \Delta F$, and thus, the poor overlap regime would be where all samples $\mathcal{W}_i \ll 0$ for some finite batch size of collected samples N_b. In this regime, we can see that $\operatorname{sech}(x) \sim 2e^x$, and $\operatorname{sech}(x)\,(1-\tanh(x)) \sim 4e^x$, thus we obtain as $\mathcal{W}_i\ \forall i$ becomes very negative, we have —

$$
\frac{\partial \mathbb{D}_{\mathrm{HD}}}{\partial \theta} \rightarrow -\frac{\left\langle e^{\mathcal{W}}\frac{\partial \mathcal{W}}{\partial \theta}\right\rangle_0}{\left\langle e^{\mathcal{W}}\right\rangle_0} = -\frac{\frac{\partial}{\partial \theta}\left\langle e^{\mathcal{W}}\right\rangle_0}{\left\langle e^{\mathcal{W}}\right\rangle_0}
\tag{S19}
$$

Interestingly, the Jarzynski equality tells us that $\left\langle e^{\mathcal{W}}\right\rangle_0 = 1$, which means that in the $\mathcal{W}_i \ll 0\ \forall i$ regime $\partial \mathbb{D}_{\mathrm{HD}}/\partial\theta \rightarrow 0$. This is generally not true for $\partial \mathbb{D}_{\mathrm{BD}}/\partial\theta = -\frac{\partial}{\partial \theta}\left\langle e^{\mathcal{W}/2}\right\rangle_0 / \left\langle e^{\mathcal{W}/2}\right\rangle_0$, due to the factor of 1/2 in the exponential. While interesting, this fact does not seem to be very relevant in practice due to the ensemble average $\left\langle e^{\mathcal{W}}\right\rangle_0$ being essentially impossible to converge for a finite number of samples.

In practice, within a stochastic mini-batch optimization step, we are estimating these gradients as the following finite sample averages —

$$
\frac{\partial \mathbb{D}_{\mathrm{BD}}}{\partial \theta} \approx \frac{1}{N_b}\sum_{i=1}^{N_b}\left[-\frac{\frac{1}{2}e^{\frac{\mathcal{W}_i}{2}}}{\left\langle e^{\frac{\mathcal{W}}{2}}\right\rangle_0}\right]\frac{\partial \mathcal{W}_i}{\partial \theta} = \frac{1}{N_b}\sum_{i=1}^{N_b} w_i^{\mathrm{BD}}\frac{\partial \mathcal{W}_i}{\partial \theta}
\tag{S20}
$$

$$
\frac{\partial \mathbb{D}_{\mathrm{HD}}}{\partial \theta} \approx \frac{1}{N_b}\sum_{i=1}^{N_b}\left[-\frac{e^{\mathcal{W}_i}}{\left\langle e^{\mathcal{W}}\right\rangle_0}\right]\frac{\partial \mathcal{W}_i}{\partial \theta} = \frac{1}{N_b}\sum_{i=1}^{N_b} w_i^{\mathrm{HD}}\frac{\partial \mathcal{W}_i}{\partial \theta}
\tag{S21}
$$

where w_i refers to a loss-specific weight applied to the derivative of the dissipation with respect to some mapping parameters (this derivative will be large at low overlap). Thus, we

can measure the effective number of samples contributing to our gradient estimate using the Kish Effective Sample Size $\left(N_{\text{eff}} = \left[\sum_i w_i\right]^2 / \sum_i w_i^2\right)$[7] —

$$\frac{N_{\text{eff}}^{\text{BD}}}{N_{\text{eff}}^{\text{HD}}} = \frac{\left(\sum_i e^{\frac{\mathcal{W}_i}{2}}\right)^2 \left(\sum_i e^{2\mathcal{W}_i}\right)}{\left(\sum_i e^{\mathcal{W}_i}\right)^3} \geq 1 \tag{S22}$$

where $N_{\text{eff}}^{\text{BD}} \geq N_{\text{eff}}^{\text{HD}}$ is given directly by Hölder's inequality.[8] However, we can derive a further result if we assume that our forward work distribution is Gaussian with mean $\mu = \beta\left(\Delta F - \left\langle \tilde{W}^{(F)} \right\rangle_0\right)$ and variance σ^2. Since the number of samples is equal in all sums, we can see that —

$$\frac{N_{\text{eff}}^{\text{BD}}}{N_{\text{eff}}^{\text{HD}}} = \frac{\left\langle e^{\frac{\mathcal{W}}{2}} \right\rangle_0^2 \left\langle e^{2\mathcal{W}} \right\rangle_0}{\left\langle e^{\mathcal{W}} \right\rangle_0^3} \tag{S23}$$

Under the Gaussian assumption, these ensemble averages are moment generating functions of the form $\left\langle e^{\alpha\mathcal{W}} \right\rangle_0 = \exp\left[\alpha\mu + \frac{1}{2}\alpha^2\sigma^2\right]$, which gives —

$$\frac{N_{\text{eff}}^{\text{BD}}}{N_{\text{eff}}^{\text{HD}}} = \frac{\exp\left[3\mu + \frac{9}{4}\sigma^2\right]}{\exp\left[3\mu + \frac{3}{2}\sigma^2\right]} = e^{\frac{3}{4}\sigma^2} \tag{S24}$$

Thus, in the Gaussian approximation, where high σ^2 indicates low overlap, the BD gradient has an exponentially larger effective sample size.

S4 Considerations for Skewed Bhattacharyya Distances

We would like our alternative overlap metric (where here we are examining skewed Bhattacharyya distances (SBDs) defined in Eq. 24 of the main text) to be an upper bound on the mapped harmonic overlap $\tilde{\mathcal{H}}_{0,1}$. Generally, this means that we require (considering averages over state 0 without loss of generality) —

$$\left\langle \frac{2}{1 + e^{-\mathcal{W}}} \right\rangle_0 \leq \left\langle e^{\alpha\mathcal{W}} \right\rangle_0 \tag{S25}$$

For this to hold for all possible work distributions, we require this inequality to hold point-wise for all possible values of the dissipation $\mathcal{W}$. Thus, we want to consider the function —

$$h(\alpha, \mathcal{W}) = e^{\alpha \mathcal{W}} - 2\sigma(\mathcal{W}) \tag{S26}$$

where $\sigma(x) = 1/(1+e^{-x})$ is again the sigmoid function. We can immediately see that $h(\alpha, 0) = 0$. Differentiating with respect to the dissipation, we get —

$$\frac{\partial h}{\partial \mathcal{W}} = \alpha e^{\alpha \mathcal{W}} - 2\sigma(\mathcal{W})\left(1 - \sigma(\mathcal{W})\right) \tag{S27}$$

$$\frac{\partial^2 h}{\partial \mathcal{W}^2} = \alpha^2 e^{\alpha \mathcal{W}} - 2\sigma(\mathcal{W})\left(1 - \sigma(\mathcal{W})\right)\left(1 - 2\sigma(\mathcal{W})\right) \tag{S28}$$

Since $\sigma(0) = 1/2$, we have that at $\mathcal{W} = 0$, $\partial h/\partial \mathcal{W} = \alpha - 1/2$. Thus, unless $\alpha = 1/2$ and $h''(1/2, 0) > 0$, h is guaranteed to be negative at some point in a neighborhood of $\mathcal{W} = 0$. Checking that $h''(1/2, 0) = 1/4$, and noting that $\alpha = 1/2$ has to provide a global point-wise bound due to the fact that the geometric mean is at least as large as the harmonic mean, we can see that $\alpha = 1/2$ uniquely bounds the harmonic overlap metric from above for any arbitrary work distribution.

S5 Derivation of the 2D MNF Jacobian

In this section we will expand on the radial and orientational transformation we apply with our 2D MNF and derive the Jacobian of this transformation. If $\mathbf{r}_{\mathrm{ion}}$ is the inserted ion position and the coordinates of water molecule i are given by $(\mathbf{r}_{\mathrm{O,i}}, \mathbf{r}_{\mathrm{H1,i}}, \mathbf{r}_{\mathrm{H2,i}})$, we first apply a radial mapping to the molecule, where the ion-oxygen distance is mapped as in Eq. 17 of the main text and the hydrogens are moved rigidly — $(\mathbf{r}_{\mathrm{O,i}}, \mathbf{r}_{\mathrm{H1,i}}, \mathbf{r}_{\mathrm{H2,i}}) \to \left(f(\mathbf{r}_{\mathrm{O,i}}, \mathbf{r}_{\mathrm{ion}}), \mathbf{r}'_{\mathrm{H1,i}}, \mathbf{r}'_{\mathrm{H2,i}}\right)$, where $\mathbf{r}'_{\mathrm{Hj,i}} = f(\mathbf{r}_{\mathrm{O,i}}, \mathbf{r}_{\mathrm{ion}}) + (\mathbf{r}_{\mathrm{Hj,i}} - \mathbf{r}_{\mathrm{O,i}})$. The logarithm of the Ja-

cobian determinant for this transformation is equivalent to Eq. 18 in the main text applied to only the ion-oxygen distances.

After this radial mapping step, we can transform our water coordinate into $(\mathbf{R}_i, \mathbf{a}_i, \mathbf{b}_i)$, where $\mathbf{R}_i = \mathbf{r}'_{\mathrm{O},i} - \mathbf{r}_{\mathrm{ion}}$ is the modified ion-oxygen vector, $\mathbf{a}_i = \mathbf{r}'_{\mathrm{H2},i} - \mathbf{r}'_{\mathrm{H1},i}$ is the H-H vector, and $\mathbf{b}_i = \frac{1}{2}(\mathbf{r}'_{\mathrm{H1},i} + \mathbf{r}'_{\mathrm{H2},i}) - \mathbf{r}'_{\mathrm{O},i}$ is the H-O-H bisector. The Jacobian determinant of this transformation is 1. We want to transform the vectors $\mathbf{a}_i$ and $\mathbf{b}_i$ into spherical coordinates $\mathbf{b}_i \to (\|\mathbf{b}_i\|, \gamma_i, \xi_i)$ and $\mathbf{a}_i \to \left(\|\mathbf{a}_i\|, \gamma_i^{(b)}, \xi_i^{(b)}\right)$, where we define —

$$\gamma_i = \arccos\left(\frac{\mathbf{R}_i \cdot \mathbf{b}_i}{\|\mathbf{R}_i\| \, \|\mathbf{b}_i\|}\right) \tag{S29}$$

as the angle between the ion-O vector and the H-O-H bisector, and ξ_i is the azimuthal angle of $\mathbf{b}_i$ about $\mathbf{R}_i$. $\left(\gamma_i^{(b)}, \xi_i^{(b)}\right)$ are the spherical angles of the vector $\mathbf{a}_i$ about $\mathbf{b}_i$ as the polar axis, with $\gamma_i^{(b)} = \pi/2$, by definition in our rigid water model. After the mapping from $r \to r' = \psi(r)$, our 2D mapping modifies the angle γ_i according to the mapping $\gamma \to \gamma' = g(\gamma|r)$, where g is conditioned on the ion-oxygen distance r prior to any transformation, by applying the Rodrigues rotation formula to the $\mathbf{a}_i$ and $\mathbf{b}_i$ vectors —

$$\begin{aligned} \mathbf{u}'_i = \mathbf{u}_i \cos(\delta) + (\mathbf{v}_i \times \mathbf{u}_i) \sin(\delta) \\ + \mathbf{v}_i (\mathbf{v}_i \cdot \mathbf{u}_i)(1 - \cos(\delta)) \end{aligned} \tag{S30}$$

where $\mathbf{v}_i = (\mathbf{R}_i \times \mathbf{b}_i) / \|\mathbf{R}_i \times \mathbf{b}_i\|$ is the axis of rotation, $\delta = \gamma' - \gamma$, and $\mathbf{u}$ refers to vectors $\mathbf{a}$ or $\mathbf{b}$. This rotation leaves the angles ξ_i and $\xi_i^{(b)}$ unaffected. Since this transformation is applied to the radially transformed water molecule, the log-Jacobian determinant of the entire transformation is the sum of the log-Jacobian determinants for each step. This is clearly seen from the fact that the Jacobian of the transformation $(r, \gamma) \to (r', \gamma')$ is lower

triangular. Thus, the total log-Jacobian determinant is given by —

$$\begin{aligned}\ln|\det\,\mathcal{J}(x)| = &\sum_{i\in \mathrm{O}} \ln\left[\frac{\psi(r_{i,\mathrm{ion}})^2\psi'(r_{i,\mathrm{ion}})}{r_{i,\mathrm{ion}}^2}\right] \\ &+ \sum_{i\in \mathrm{O}} \ln\left[\frac{\sin(g(\gamma_i|r_{i,\mathrm{ion}}))g'(\gamma_i|r_{i,\mathrm{ion}})}{\sin(\gamma_i)}\right]\end{aligned} \tag{S31}$$

which is trivial to calculate in terms of computational expense for any proposed 2D mapping.

S6 Details of 1D and 2D MNFs

To implement our mapping functions ψ and g, we use rational quadratic splines as proposed by Durkan *et al.*[9] The parameter set of a 1D spline is composed of a collection of spline knot point locations ($\{(x_k, y_k)\}_{k=1}^{N_p}$) and associated slopes at those knot points ($\{d_k\}_{k=1}^{N_p}$), where N_p is the number of knots in our spline. Thus, the parameters $\boldsymbol{\theta}$ can be succinctly represented as the collection of vectors $\boldsymbol{\theta} = (\mathbf{x}, \mathbf{y}, \mathbf{d})$. For our simple 1D map, we of course have that $x = r$ and $y = r'$.

To represent our 2D mapping, we split our mapping parameters into two sets — radial mapping parameters $\boldsymbol{\theta}_r = (\mathbf{x}_r, \mathbf{y}_r, \mathbf{d}_r)$ and orientational mapping parameters $\boldsymbol{\theta}_\gamma(r) = (\mathbf{x}_\gamma, \mathbf{y}_\gamma(r), \mathbf{d}_\gamma(r))$. The orientational mapping operates on the quantity $\cos\gamma$, where $x = \cos\gamma$ and $y = \cos\gamma'$. For every conditioning value of r, the x knot positions are constant and are $N_{p,\gamma}$ evenly spaced knots within $x \in [-1, 1]$. However, the y knot positions and slopes are a function of r, where —

$$\mathbf{y}_\gamma(r) = \frac{\mathbf{y}_\gamma(r_k)\,(r_{k+1} - r) + \mathbf{y}_\gamma(r_{k+1})\,(r - r_k)}{r_{k+1} - r_k} \tag{S32}$$

where $r \in [r_k, r_{k+1}]$, r_k are points from the set of x knot locations of the radial mapping ($\mathbf{x}_r$), and we define an orientational mapping parameter set $\boldsymbol{\theta}_\gamma(r_k)$ for all k (these are the

orientational mapping parameters affected by training). Orientational mapping slopes $\mathbf{d}_\gamma(r)$ are defined by an equivalent linear interpolation expression.

For any spline parameterized by the tuple $\boldsymbol{\theta} = (\mathbf{x}, \mathbf{y}, \mathbf{d})$, we must ensure that the spline is monotonic in order to guarantee bijectivity (and thus invertability). We do this by first transforming the parameter vector to represent differences between values of consecutive knot points, $(\mathbf{x} \to \boldsymbol{\Delta}\mathbf{x}, \mathbf{y} \to \boldsymbol{\Delta}\mathbf{y})$, where $\boldsymbol{\Delta}\mathbf{x}$ is a vector of length one less than $\mathbf{x}$, $\Delta x_k = x_{k+1} - x_k$, and all differences are referenced to the point $x_0 = 0$ (and this all holds for $\boldsymbol{\Delta}\mathbf{y}$ as well). For bijectivity to hold, both x and y knot points need to be monotonically increasing, and derivatives at the knot points need to be nonnegative. In addition, for our splines that act on radial distances (ψ), we need our knot points to not extend beyond the fitting limit (r_f) described in the main text (Sec. 3), so we need $x_k, y_k \in [0, r_f]$. Thus, the slopes and knot point differences get transformed as follows —

$$\Delta\tilde{x}_k = f^{(sp)}\left(\Delta x_k, \eta\right) \frac{r_f}{\delta + \sum_k f^{(sp)}\left(\Delta x_k, \eta\right)} \tag{S33}$$

$$\tilde{d}_k = f^{(sp)}\left(d_k, \eta\right) \tag{S34}$$

where Eq. S33 is applied equivalently to obtain $\Delta\tilde{y}_k$, $\delta = 10^{-8}$ is an infinitesimal offset factor to prevent divergences, and $f^{(sp)}$ refers to a scaled softplus function that is differentiable but ensures non-negativity —

$$f^{(sp)}(t, \eta) = \frac{1}{\eta} \ln\left(1 + e^{\eta t}\right) \tag{S35}$$

where we always use $\eta = 50$. The differences in knot point values are then added back to the $(x_0, y_0) = (0, 0)$ reference to obtain the parameters $\tilde{\boldsymbol{\theta}} = \left(\tilde{\boldsymbol{x}}, \tilde{\boldsymbol{y}}, \tilde{\boldsymbol{d}}\right)$, which are now guaranteed to give a bijective, differentiable function. The final modification is that the parameters (r_f, r_f) and $(L_{\max}, L_{\max})$, with slopes of 1 at each of these knot points, are appended to the parameter set $\tilde{\boldsymbol{\theta}}$, where $L_{\max}$ is the maximum box length seen in states 0 or 1. This ensures

that any pairwise distance seen by the spline is within its domain, and applies the identity map for $r > r_f$.

For our orientational mapping function (g) parameters, an identical procedure is applied, except the reference point for knot point differences is $(x_0, y_0) = (-1, -1)$, and r_f is replaced with 2 in our transformation equation (Eq. S33). For the appended points on the radial spline, the associated orientational maps are set to be an identity mapping.

S6.1 MNF Hyperparameters

The number of radial mapping knot points reported (N_p^r) is the number of points used within the fitting limit, the overall spline with the appended points has $N_p^r + 2$ knots. The number of knot points for each orientational spline at each r knot point is referred to as N_p^γ. For all test cases, we use $N_p^r = 50$. For the electrolyte test cases where we have a 2D mapping, we use $N_p^\gamma = 15$. All mappings are trained using stochastic mini-batch gradient descent implemented using JAX and using the Adam optimizer.[10,11] For all optimizations we use a mini-batch size of 128 configurations, a learning rate of 5×10^{-5}, and we train for 1000 epochs. For LJ test cases, we set $r_f = 7$ Å and for electrolyte test cases we set $r_f = 6$ Å.

S7 Accelerating MNF Training

As described in the main text, our radial mapping is constrained to only be "non-identity" up to some radius r_f, which we refer to as a "fitting limit". This creates two regions — an "interior" (I) region where atomic coordinates are perturbed by the mapping, and an "exterior" (E) region where the atoms are untouched by the mapping. While computing mapped works during training, we need to calculate the potential energy function of state 1 applied to a mapped configuration sampled from state 0, which is written as $U_1(\phi(x, \boldsymbol{\theta}))$.

We can decompose this mapped energy as —

$$U_1(\phi(x,\boldsymbol{\theta})) = \sum_{i\in \mathrm{I}}\sum_{j>i} u_{ij}^{(1)}(\phi(\mathbf{r}_i,\mathbf{r}_j,\mathbf{r}_{\mathrm{ins}},\boldsymbol{\theta})) + \sum_{i\in \mathrm{E}}\sum_{j\in \mathrm{E},\, j>i} u_{ij}^{(1)}(r_{ij}) \tag{S36}$$

which is given in Eq. 32 of the main text, and again we assume that the "Inner" atoms are given the first N_I indices. It is clear that the second term is independent of the mapping parameters. Thus, to fully resolve $U_1(\phi(x,\boldsymbol{\theta}_t))$ at each training step t, we are only required to compute the quantity —

$$\Delta U_1^{(t)}(x,\boldsymbol{\theta}) = U_1(\phi(x,\boldsymbol{\theta}_t)) - U_1(x) = \sum_{i\in \mathrm{I}}\sum_{j>i}\{u_{ij}^{(1)}(\phi(\mathbf{r}_i,\mathbf{r}_j,\mathbf{r}_{\mathrm{ins}},\boldsymbol{\theta}_t)) - u_{ij}^{(1)}(r_{ij})\} \tag{S37}$$

which is the change in energy induced by the mapping relative to the identity map. For each sample, the E-E term will be independent of the mapping parameters. For the LJ test cases, where we only have non-Coulombic interactions in the form of an LJ potential, this energy difference becomes —

$$\Delta U_1^{(t)}(x,\boldsymbol{\theta}) = \sum_{i\in \mathrm{I}}\sum_{j>i}\left[u_{ij}^{(\mathrm{LJ})}(\phi_t(r_{ij})) - u_{ij}^{(\mathrm{LJ})}(r_{ij})\right] \tag{S38}$$

where $\phi_t(r_{ij}) = \phi(r_{ij},\boldsymbol{\theta}_t)$ is the perturbed radial distance between atoms i and j determined by the application of the 1D MNF with parameter set $\boldsymbol{\theta}_t$. For the electrolyte case, where we have Ewald sums, this becomes —

$$\begin{aligned}\Delta U_1^{(t)}(x,\boldsymbol{\theta}) = &\sum_{i\in \mathrm{I}}\sum_{j>i}\left[u_{ij}^{(\mathrm{SR})}(\phi_t(r_{ij})) - u_{ij}^{(\mathrm{SR})}(r_{ij})\right] \\ &+ \frac{1}{2V}\sum_{\mathbf{k}\neq 0}\frac{4\pi}{k^2}e^{-\frac{k^2}{4\alpha^2}}\left(2\mathrm{Re}\{\rho^*(\mathbf{k})\Delta\rho(\mathbf{k})\} + |\Delta\rho(\mathbf{k})|^2\right)\end{aligned} \tag{S39}$$

where α is the typical Ewald splitting parameter, $\mathbf{k}$ is a lattice vector in reciprocal space, $\rho(\mathbf{k})$ is the Fourier transform of the charge density,[12] $\phi_t(r_{ij}) = \phi(r_{ij},\boldsymbol{\theta}_t)$ the perturbed radial distance between atoms i and j determined by the application of the 2D MNF with

parameter set $\boldsymbol{\theta}_t$, and where we have that —

$$u_{ij}^{(\mathrm{SR})}(r_{ij}) = u_{ij}^{(\mathrm{LJ})}(r_{ij}) + \frac{q_i q_j}{4\pi\epsilon_0 r_{ij}}\mathrm{erfc}(\alpha r_{ij}) \tag{S40}$$

$$\Delta\rho(\mathbf{k}) = \sum_{i\in\mathrm{I}} q_i \left[e^{i\mathbf{k}\cdot\phi_t(\mathbf{r}_i)} - e^{i\mathbf{k}\cdot\mathbf{r}_i}\right] \tag{S41}$$

Assuming we have $N = N_\mathrm{I} + N_\mathrm{E}$ atoms and N_k reciprocal space vectors for our Ewald sum, this approach makes the short-range energy change calculation at each training step $\mathcal{O}(N_\mathrm{I} \cdot N)$ and the long-range energy change calculation $\mathcal{O}(N_\mathrm{I} \cdot N_k)$, which provides serious computational savings in a liquid system, especially when $r_f \approx L/4$.

S8 Comparisons between Loss Strategies

Based on the theoretical discussions in the main text, it is interesting to directly compare the performance of the KLD, BD, HD, and BD-HD training approaches on some model systems and test our theoretical claims. The first test system that we consider is a more difficult version of the pure LJ system examined in the main text, now at conditions of $\rho^* = 1.1$ and $T^* = 2.5$, which is a liquid, almost at crystallization conditions. After initial sample collection, the initial Bennett Acceptance Ratio (BAR) estimate (on the training set) fails in this system, estimating the chemical potential to be $\beta\hat{\mu}^{0\to1} \approx 27$. This chemical potential estimate is significantly biased compared to the ground truth value we computed using 10 linearly spaced windows — $\beta\mu^{0\to1} = 12.13 \pm 0.04$. The identity mapping applied to the evaluation set gives $\beta\hat{\mu}^{0\to1} = 19 \pm 4$. We then trained 1D MNFs (for 2000 epochs with each loss using a learning rate of 2×10^{-5}, with all other hyper-parameters as described in Sec. S6.1) to accelerate the chemical potential calculation for this system using all four training approaches above.

Fig. S1 shows plots of the variance in our targeted BAR estimate (evaluated on the training data using Eq. 5 in the main text, with a bootstrapping fallback if this estimate

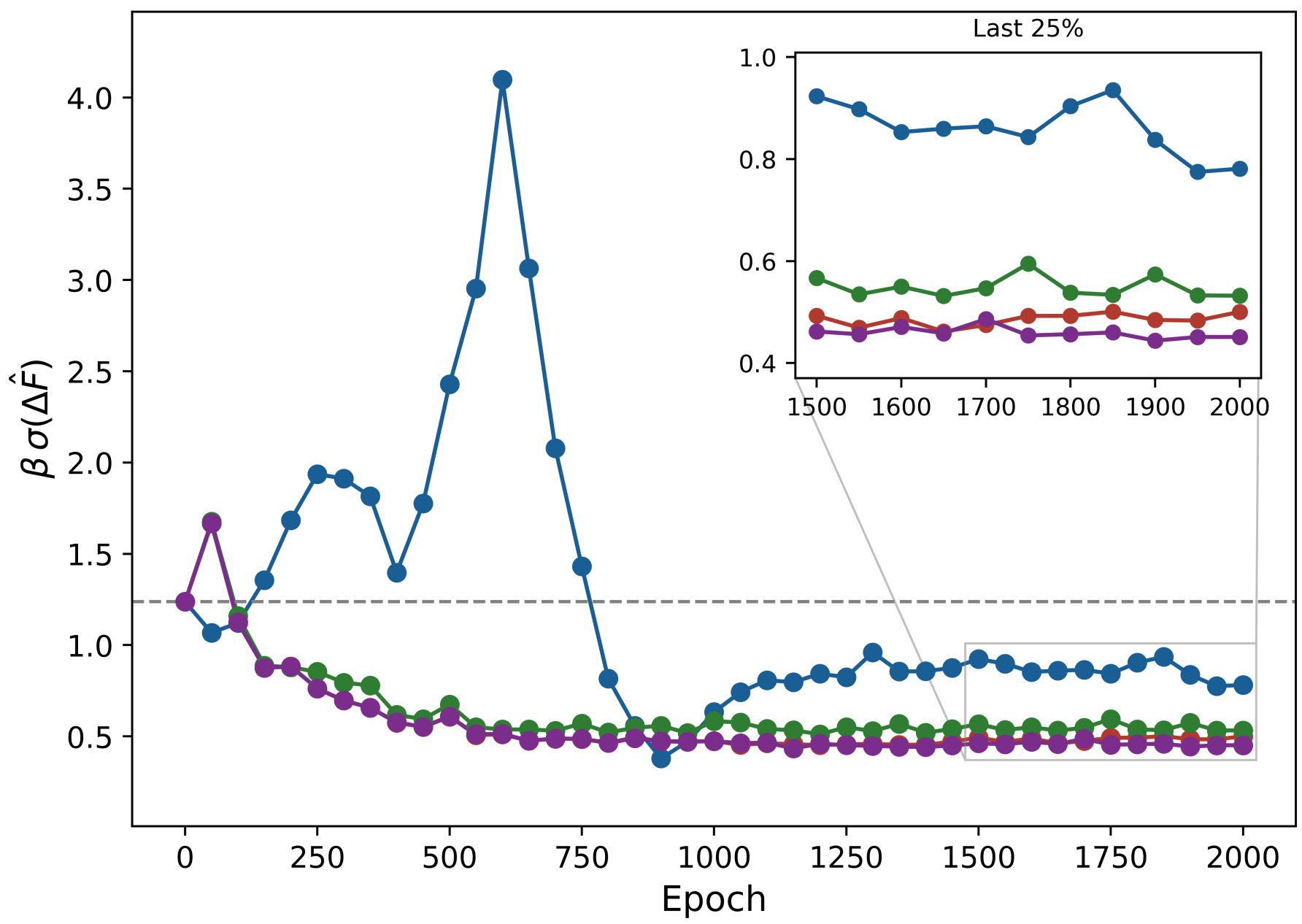

Figure S1: **Standard error over time of targeted chemical potential estimates for a Pure LJ system ($\rho^* = 1.1$ and $T^* = 2.5$) using various training strategies.** The standard error of the targeted chemical potential evaluated on the training set for the mappings trained using the KLD, BD, HD, and BD-HD losses are given by the blue, red, green, and purple lines, respectively. The inset zooms in on the last 25% of training steps.

is undefined using 500 bootstraps) as a function of training time. We can see that the targeted estimate using the 1D MNF trained on the KLD (blue line) performs the worst, and does not lead to an accurate and precise chemical potential estimate when applied to our evaluation set, giving an estimate of $\beta\hat{\mu}^{0\to1}_{\mathrm{KL}} = 10.9 \pm 1.5$. This provides further evidence for the pathologies hypothesized in Sec. 2.4.2 of the main text. As seen in the zoomed in inset, the BD (red), HD (green), and BD-HD (purple) training strategies all achieve lower standard errors on the training set than the KLD. The higher resulting error of the HD training on the training data (compared to the BD-HD training) can be explained by the dependence of this loss on the bias in the initial free energy estimate as described in the main text and in Sec. S3.1. Interestingly, all three estimates provide essentially equivalent results on the evaluation set — the HD optimized mapping provides an estimate of $\beta\hat{\mu}^{0\to1} = 11.7 \pm 0.5$, while the BD and BD-HD optimized mappings provide estimates of $\beta\hat{\mu}^{0\to1} = 11.8 \pm 0.5$. This points to the fact that the theoretical distinctions between these losses are not enough to

impact performance on the evaluation set for this simple and low-dimensional optimization problem.

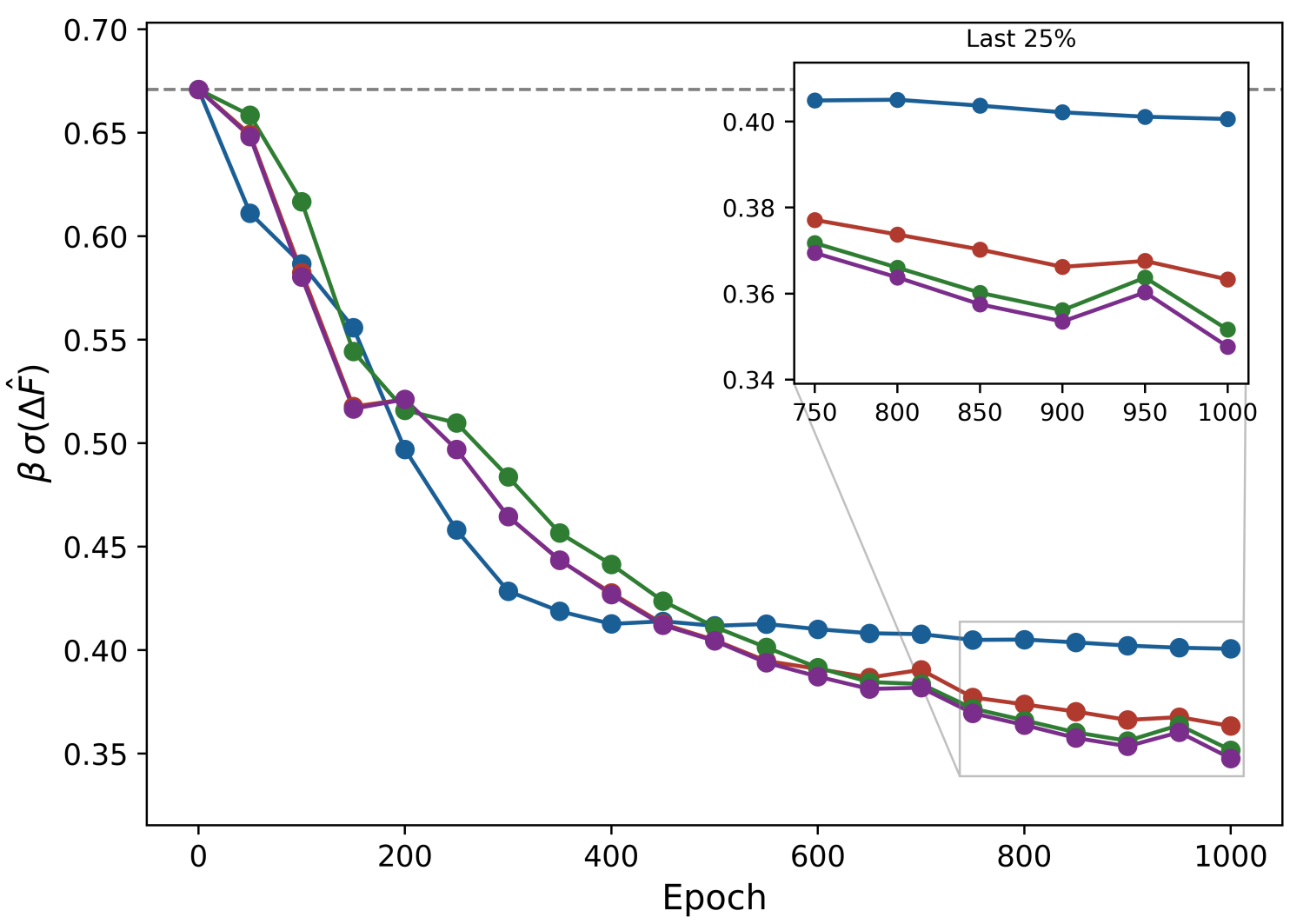

Figure S2: **Standard error over time of targeted charging free energy estimates for Na^+ in water ($\phi = 0.0 \rightarrow \phi = 0.5$) using various training strategies.** The standard error of the targeted charging free energy estimate evaluated on the training set for the mappings trained using the KLD, BD, HD, and BD-HD losses are given by the blue, red, green, and purple lines, respectively. The inset zooms in on the last 25% of training steps.

We also wanted to examine the differences between these losses when training a 2D MNF to accelerate the calculation of the charging free energy of Na^+ in water. Here we are accelerating the calculation of the free energy difference in a coarse charging window between $\phi = 0.0$ and $\phi = 0.5$ by training a mapping with each of the 4 loss strategies (using a learning rate of 2×10^{-5}, with all other hyper-parameters as described in Sec. S6.1). The initial BAR estimate on the training samples $\beta\hat{\mu}^{0\to1} = -30.9 \pm 0.7$ is not biased compared to the ground truth estimate of $\beta\mu^{0\to1} = -30.90 \pm 0.03$ (which we computed using 10 evenly spaced windows in the charging parameter ϕ). Applied to the evaluation set, the identity mapping gives an estimate of $\beta\hat{\mu}^{0\to1} = -30.5 \pm 1.0$. The standard error in the BAR estimate of the free energy (evaluated on the training data using Eq. 5 in the main text, with a bootstrapping fallback if this estimate is undefined using 500 bootstraps) is plotted as a function of training time in Fig. S2. Due to the lack of bias in the initial free energy estimate, we can see that the HD (green) and BD-HD (purple) losses have very similar behavior towards the end of

training. Here we also see that the KLD training (blue) shows the worst performance on the training data, although it still provides comparable performance to the other losses in this case. Interestingly, all four losses are hard to distinguish when applied to the evaluation set, with the KLD trained mapping providing an estimate of $\beta\hat{\mu}^{0\to 1} = -30.9 \pm 0.4$, and the BD, HD, and BD-HD trained mappings providing an estimate of $\beta\hat{\mu}^{0\to 1} = -30.7 \pm 0.4$. This equivalent performance on the evaluation set may indicate that the overlap targeting mappings may be overfitting to the training data, and perhaps in the future early-stopping techniques should be applied for the training of more complex MNFs. The success of the KLD on this problem (but failure on the pure LJ test case) is the result of the symmetry between the forward and reverse work distributions for this charging test case — minimizing the distance between the means happens to align with increases in overlap here. However, the significantly worse performance of the KLD on particle insertion tasks (as seen in the LJ examples), leads to the conclusion that using the other loss schemes is almost always preferable.

Table S3: **Effective numbers of samples contributing to the gradients of the BD and HD loss functions at low overlap conditions**. Effective sample sizes for both losses are computed prior to training for both of the test cases examined above. The total number of samples is $N_{\text{ind}} = 3334$ for the LJ case and $N_{\text{ind}} = 4445$ for the electrolyte case.

**Test Case**	**Loss**	$N_{\text{eff}}^{(F)}(\nabla\mathcal{W})$	$N_{\text{eff}}^{(R)}(\nabla\mathcal{W})$
Pure LJ	BD	2.6	390.8
	HD	1.7	15.9
Elec-Charge	BD	18.8	12.0
	HD	4.2	4.9

We also confirm that at low overlap, the BD has more robust gradient behavior compared to HD, as predicted in Sec. S3.2. We do this by numerically confirming we are in the low overlap regime ($\text{sech}(x) \approx 2e^x$, and $\text{sech}(x)\,(1 - \tanh(x)) \approx 4e^x$) in the two cases considered above. In Table S3 we report the effective number of samples contributing to the average gradient of each loss in both cases, showing that the BD loss consistently has a higher number of contributing samples compared to HD. It is worth noting that neither of these effective

sample size differences adhere to the prediction of the Gaussian work distribution model, indicating some non-Gaussianity in our work distributions. In our test cases, this reduced robustness of the HD gradient at low overlap did not lead to serious issues in the actual MNF optimizations — the HD was always able to lead to effective mappings. However, we suspect that for more complicated MNFs with more parameters, where the optimization landscape is not as simple, these differences in the robustness of the gradient will play a larger role in achieving stable optimizations.

S9 Comparison between MNF-based Acceleration and Windowing

We seek to examine how MNFs can enhance or replace existing strategies for computing the chemical potential of a dense liquid. We do this by taking the typical calculation strategy — treating the inserted particle interactions with the λ-dependent soft-core LJ potential given in Eq. S1 and dividing the particle insertion free energy calculation into a series of λ windows — and comparing it to its MNF-accelerated counterpart. We refer to the standard (non-MNF-accelerated) procedure as "soft-core + windowing." For both of the pure systems of 216 LJ particles considered in the main text (at conditions $\rho^* = 0.9, T^* = 1.2$ and $\rho^* = 1.1, T^* = 2.5$), we define four different windowing schedules $\lambda = \{0.0, 1.0\}$, $\lambda = \{0.0, 0.5, 1.0\}$, $\lambda = \{0.0, 0.3, 0.7, 1.0\}$, and $\lambda = \{0.0, 0.2, 0.5, 0.8, 1.0\}$ with one, two, three, and four windows respectively. We define the inserted particle's potential energy at each λ point using Eq. S1 with $n = 1$ and $C = 1$. We then calculate the standard error in the chemical potential estimate (computed using bootstrapping) for both a typical "soft-core + windowing" estimate for a given schedule and its MNF-accelerated counterpart (a 1D radial map acceleration applied to the free energy estimate within each window). We split each trajectory at each λ point into training and evaluation sets of 1667 configurations each, and

only the evaluation configurations were used to compute free energy differences in all cases. The results for this analysis are shown in Fig. S3.

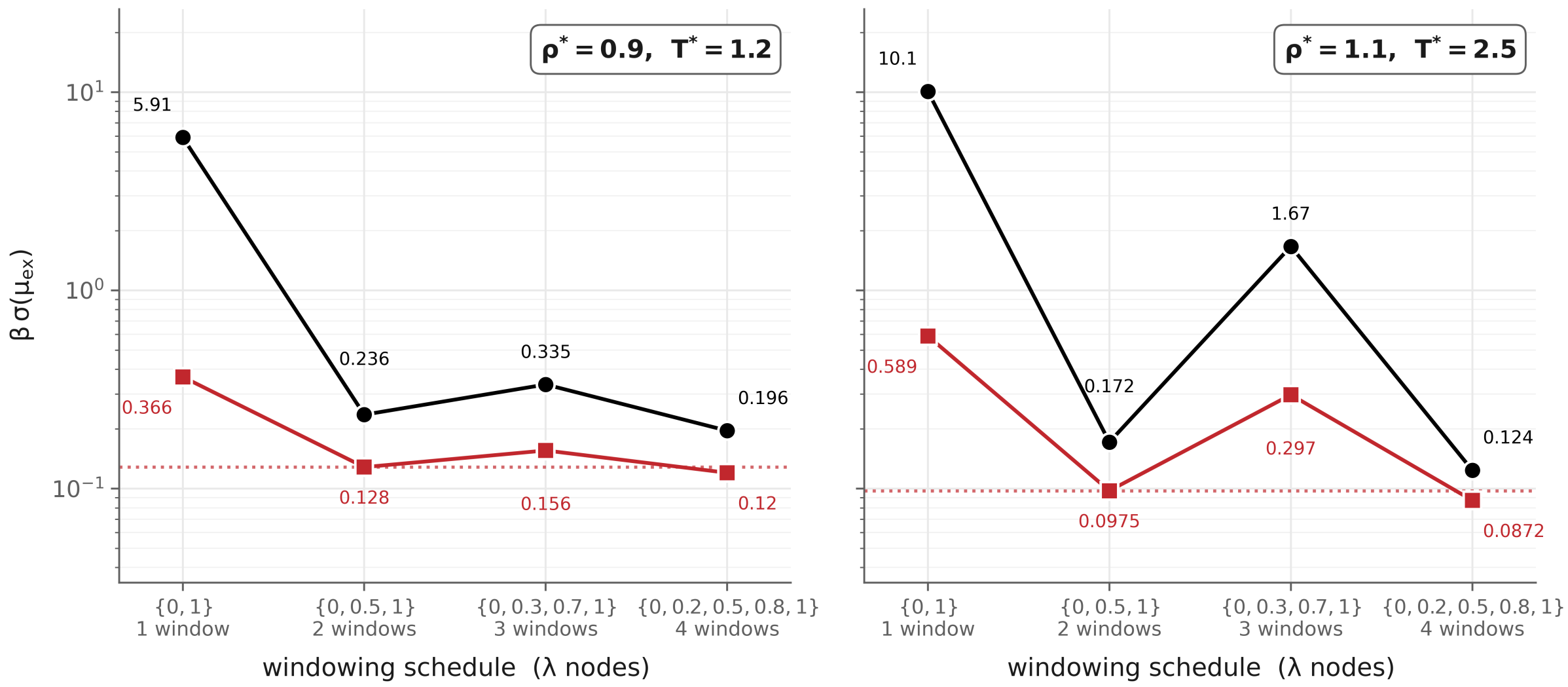

Figure S3: **Comparison between targeted free energy estimation with MNFs and a standard strategy of windowing and applying a soft-core potential.** The standard error ($\beta\sigma\left(\mu_{\text{ex}}\right)$) as a function of the windowing schedule in non-targeted calculations is shown by the black lines and points, while the standard error of MNF-accelerated estimates is shown by the red lines and points. A horizontal red dotted line through the MNF-accelerated, two-window estimate is added to show its superiority over all non-targeted estimates.

These results have several interesting implications for our method. For example, we see that in both test cases, the one-window, MNF-accelerated estimate is worse in standard error than the unmapped estimate using two windows, even when accounting for the added expense of an extra simulation in the two-window case (in both cases the standard two-window calculation requires 1.5 times the amount of sampling while achieving an at least two times lower variance). More generally, when the windowing schedule is well chosen, our mapped estimate using that schedule provides an acceleration of $\tilde{n} \approx 2 - 3$ relative to the standard estimate. However, for poorly chosen or coarse (one-step) windowing schemes, our method can provide a 1-2 order of magnitude boost in sample efficiency relative to the standard approach. Interestingly, we can see that for both systems, the standard three-window estimate is worse than the two-window estimate, with the one-window, MNF-accelerated estimate actually out-performing the standard three-window estimate in the higher density test case.

This is explained by the fact that the error in the estimate is largely determined by the error in the free energy estimate within the window with the worst overlap. Our results show that in both systems the λ-dependent path through phase space, determined by the form of the soft-core potential, contains a region of high curvature in the free energy profile concentrated near $\lambda \approx 0.3 - 0.5$ for $\rho^* = 0.9$ and near $\lambda \approx 0.4 - 0.6$ for $\rho^* = 1.1$. The two- and four-window schedules both place an intermediate state at $\lambda = 0.5$, which lies within this region and thus divides it between two windows. In contrast, the three-window schedule has intermediate states at $\lambda = 0.3$ and $\lambda = 0.7$, meaning the entire difficult portion of the path has to be handled by this single window. From this, we conclude that adding an intermediate state improves the estimate only if it splits a difficult region in the free energy profile rather than causing a single window to fully contain it.

This highlights the fact that the effectiveness of windowing depends on our knowledge of the geometry of our chosen windowing path, which would guide effective placement of intermediate states. There is no way to know *a priori* where these difficult regions along the path occur, since the identification of these regions requires an expensive scan of the λ-parameterized path using many narrow windows. We note that the location of this difficult region in the free energy profile is a function of both the soft-core potential parameters and the thermodynamic state, where the latter is directly seen by the fact that its location shifts between our two test cases.

These results bolster the advantage our method provides since our 1D radial mapping led to a reduction in error relative to the standard estimates for all windowing schedules, and in both test cases, the MNF-accelerated, two-window estimate out-performed all standard estimates (even those with four windows), as shown by the horizontal dotted line in Fig. S3. Overall, our method has the capacity to allow a one-step estimate of a chemical potential to out-perform standard windowing techniques in some cases, and it provides a reduction in error for a free energy estimate between any two states at negligible cost relative to sampling, independent of any knowledge about the geometry of paths connecting the two states

in question. Most importantly, the relative improvement our method provides is greatest precisely when the windowing schedule is poorly chosen, which is exactly the situation that knowledge of the path geometry would be required to avoid.

S10 Examples of 1D and 2D MNFs

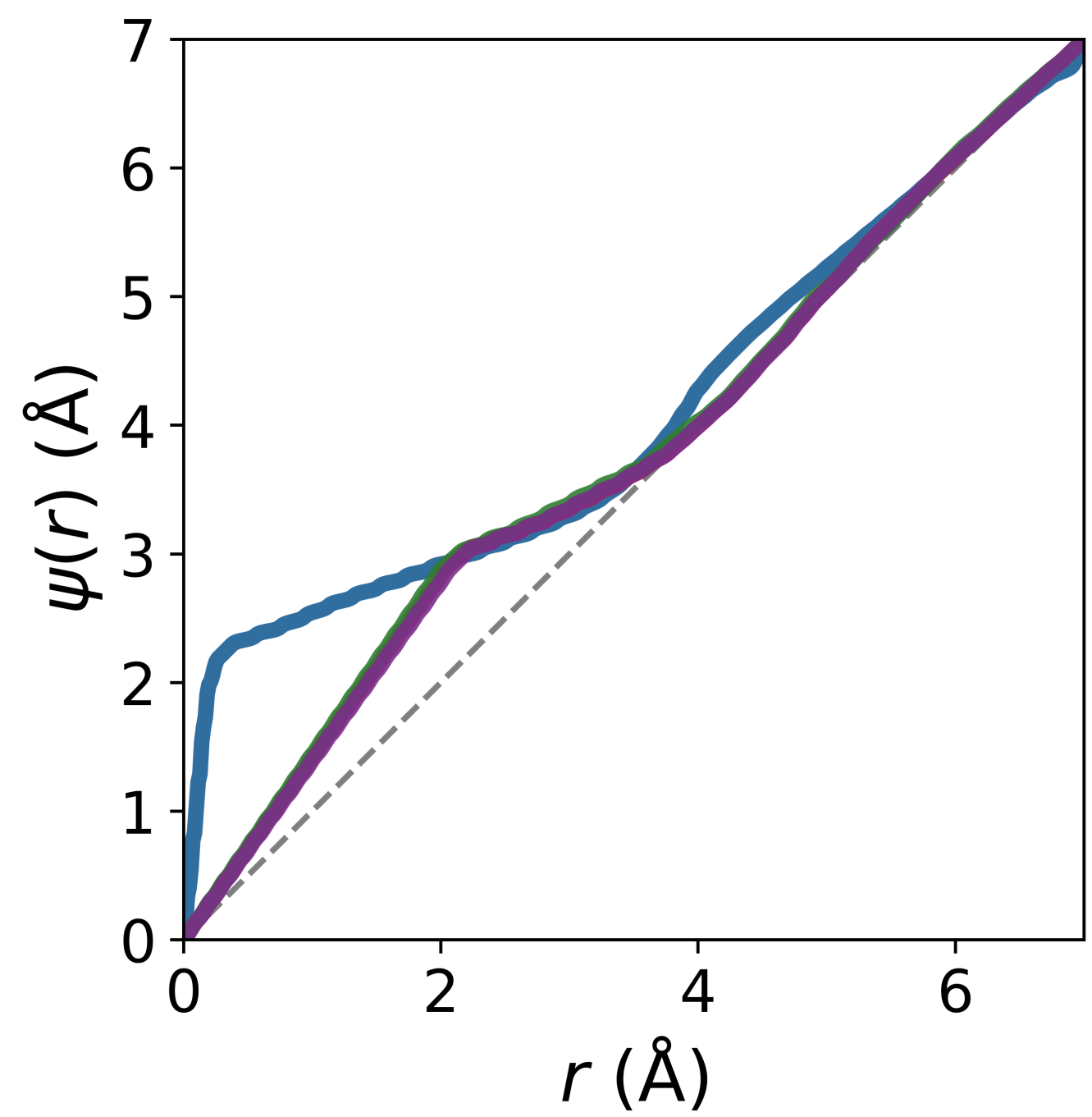

Figure S4: **Trained 1D MNFs for the challenging Pure LJ system ($\rho^* = 1.1$, $T^* = 2.5$)**. Here optimized mappings ($\psi(r)$) obtained using the KLD, BD, HD, and BD-HD losses are given by the blue, red, green, and purple lines and symbols, respectively (red and purple essentially overlap).

We provide examples of what the trained ψ and g functions for our 1D and 2D MNFs look like. Fig. S4 shows the results of training a 1D MNF for our challenging pure LJ system ($\rho^* = 1.1$, $T^* = 2.5$) for all four losses. We can see that the BD (red), HD (green), and BD-HD (purple) training strategies all converge to very similar ψ functions, with BD and BD-HD arriving at indistinguishable mappings, while optimizing the HD loss gives a slightly different solution. On the other hand, the KLD-trained mapping (blue) is significantly different from the other three. This aligns with the training behavior seen in Fig. S1.

We also show the 2D MNF trained for the Na^+ charging test case examined above ($\phi = 0.0 \rightarrow \phi = 0.5$). Fig. S5 shows the radial mapping in the left panel and a heat map of the orientational mapping in the right panel, now only for the BD-HD training strategy. Here the radial mapping is rather subtle, but the action of the orientational mapping is more pronounced, signaling that water orientations in the hydration shell are more important for enhancing phase space overlap than radial pushing/pulling.

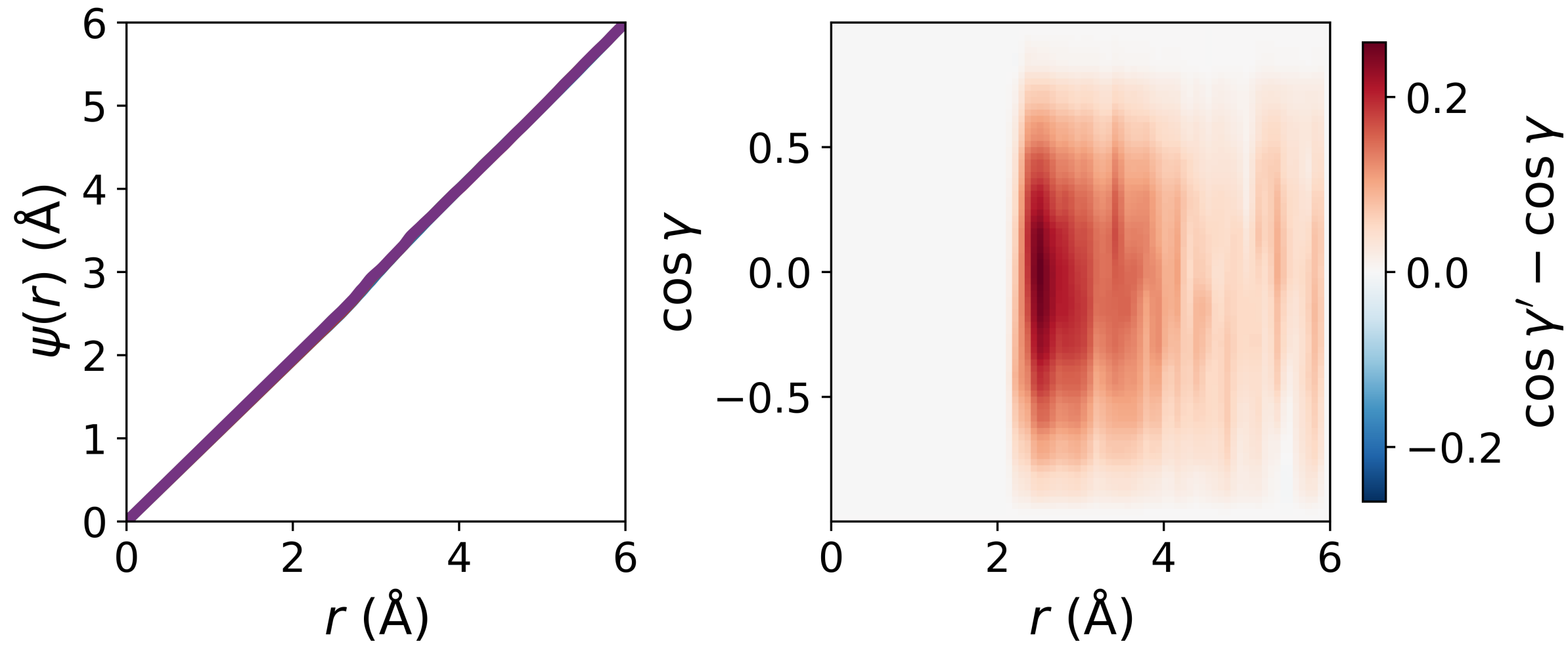

Figure S5: **Trained 2D MNF for the charging of Na^+ from $\phi = 0.0$ to $\phi = 0.5$.** The left panel shows the radial mapping trained using the BD-HD loss function. The right panel shows a heat map of differences between the optimized orientational mapping trained using the BD-HD loss function and the identity orientational mapping.

S11 Results for the Charging Free Energy of Cl^-

Here we show results for an acceleration of the calculation of the charging free energy of Cl^- in water, which is analogous to the results in Fig. 4 in the main text for Na^+.

The results are shown in Fig. S6, and show the decreased performance of our optimal mappings for the case of Cl^- hydration. At a window spacing of $\Delta\phi = 1/3$, we achieve an acceleration of only $\tilde{n} = 1.6$, while an acceleration of $\tilde{n} = 3$ was achieved for Na^+ at this spacing. This is due to the fact that our MNF construction happens to capture less of the transformations important for phase space overlap enhancement in the Cl^- system.

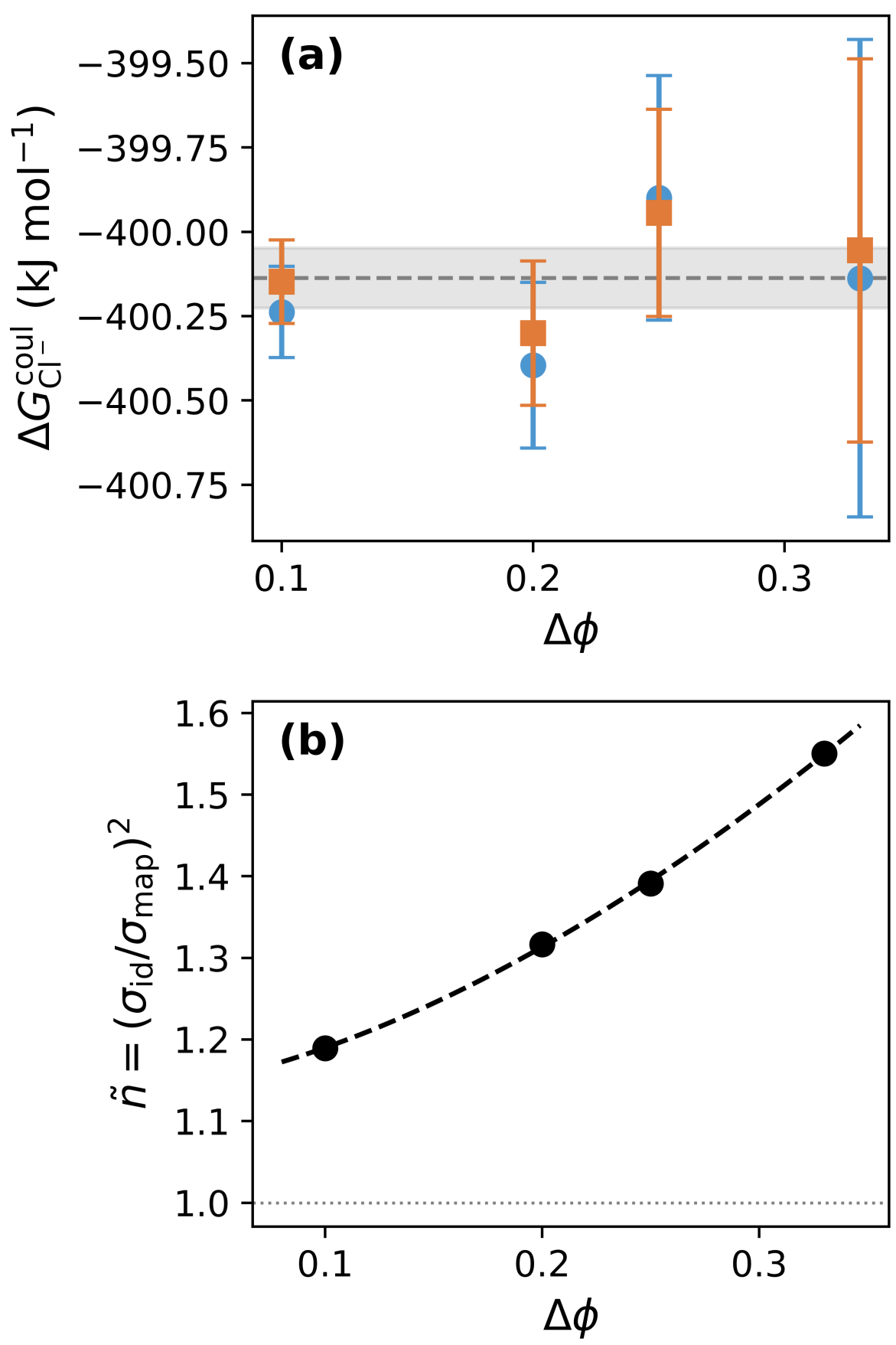

Figure S6: **Acceleration of the Charging Free Energy Calculation for Cl^-.** (a) Charging free energy (ΔG^{coul}) versus window size ($\Delta\phi$) for the identity mapping (blue) and the targeted estimate (orange) trained using the BD-HD procedure. (b) Effective sample size increase ($\tilde{n}$) versus window size ($\Delta\phi$) for the BD-HD mapping compared to identity. The dashed line represents a power law fit to the data to guide the eye.

S12 Results for the System Size Transferability of our 2D MNFs

Lastly, we show that our MNFs are transferable across system sizes. We do this by training our 2D MNF to accelerate the charging of Na^+ in a system comprised of 216 molecules of SPC/E water. We then (without re-training) apply this MNF to accelerate an equivalent calculation in a system with 648 water molecules (now also including a fully interacting ion pair in order to maintain the same concentration).

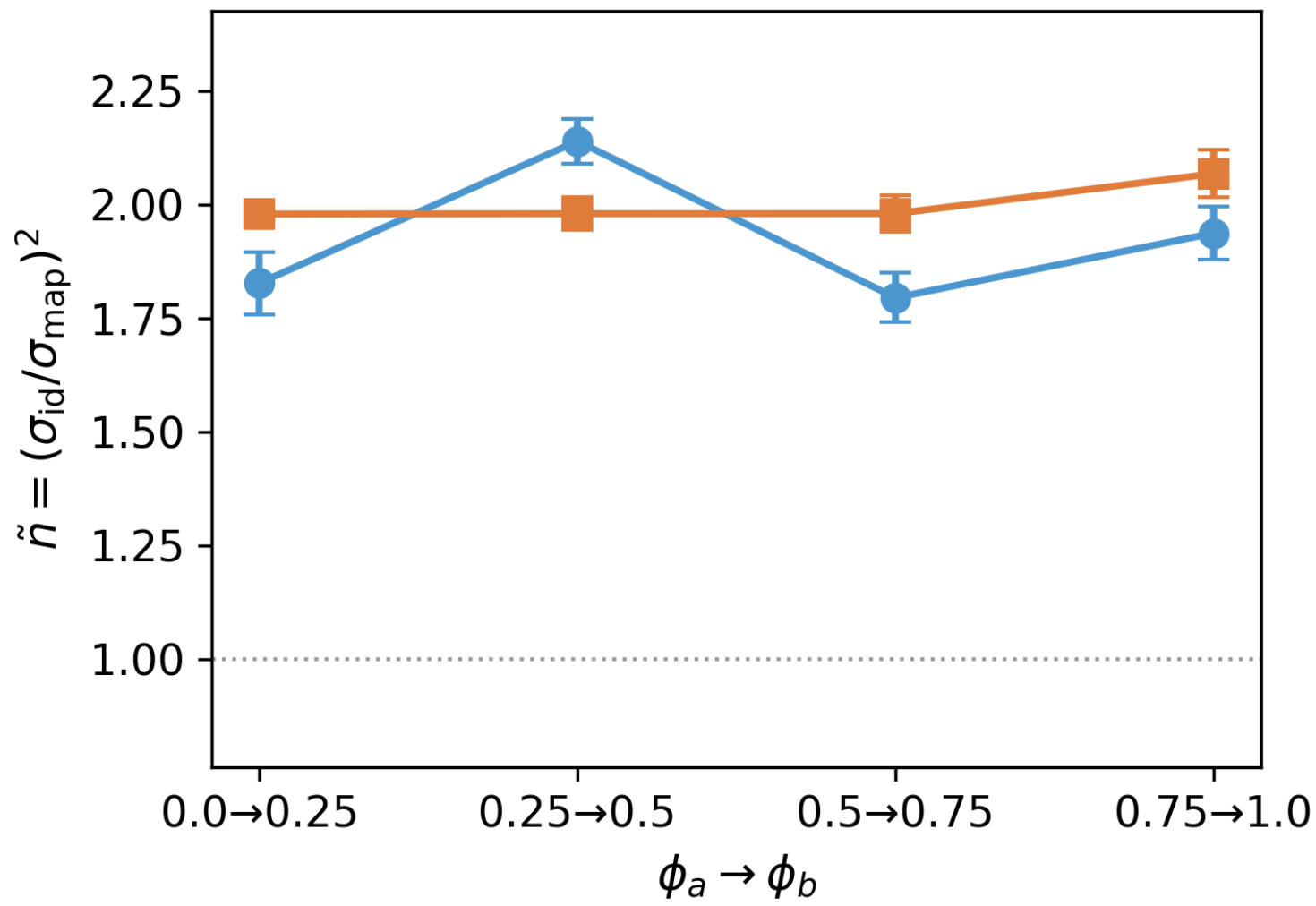

Figure S7: **Transferability of a mapping trained to accelerate the charging free energy calculation of Na^+ on a 216 water system to a larger, 648 water system**. Effective sample size increase relative to the identity mapping ($\tilde{n}$) is shown for the mapping trained on a 216 water system applied to an evaluation set of other 216 water systems (blue) and a larger, 648 water system (orange). This quantity is plotted for each of the 4 windows in a charging free energy calculation with window size $\Delta\phi = 1/4$. Error bars showing 68% confidence intervals are computed as standard errors over 5 replicate calculations over the evaluation set.

There are two technical details in how we apply the mapping trained on the 216 water system to the 648 water system. First, we need to change the value of $L_{\max}$ in the last point appended to the radial mapping (as described in Sec. S6) so that all possible pairwise distances in the larger system are guaranteed to be within the domain of the mapping. Second, we set up the mapping such that if an interacting ion enters the radius of r_f around the inserted ion (the training system has no fully interacting ion pairs while the larger system has one), we simply apply the trained ion-O radial mapping to adjust its position. The results of applying our small system mapping to accelerate the calculation of the charging free energy of Na^+ in the larger system at $\Delta\phi = 0.25$ are shown in Fig. S7. We can see that the mapping provides an essentially equivalent acceleration for the larger system, actually showing a slightly greater acceleration for some windows. This shows that our MNFs are trivially transferable to larger systems without re-training.

References

(1) Thompson, A. P.; Aktulga, H. M.; Berger, R.; Bolintineanu, D. S.; Brown, W. M.; Crozier, P. S.; Veld, P. J. I.; Kohlmeyer, A.; Moore, S. G.; Nguyen, T. D.; Shan, R.; Stevens, M. J.; Tranchida, J.; Trott, C.; Plimpton, S. J. LAMMPS-a flexible simulation tool for particle-based materials modeling at the atomic, meso, and continuum scales. *Comput. Phys. Commun.* **2022**, *271*, DOI: 10.1016/j.cpc.2021.108171.

(2) Espinosa, J. R.; Young, J. M.; Jiang, H.; Gupta, D.; Vega, C.; Sanz, E.; Debenedetti, P. G.; Panagiotopoulos, A. Z. On the calculation of solubilities via direct coexistence simulations: Investigation of NaCl aqueous solutions and Lennard-Jones binary mixtures. *J. Chem. Phys.* **2016**, *145*, DOI: 10.1063/1.4964725.

(3) Joung, I. S.; Cheatham, T. E. Determination of alkali and halide monovalent ion parameters for use in explicitly solvated biomolecular simulations. *J. Phys. Chem. B* **2008**, *112*, 9020–9041, DOI: 10.1021/jp8001614.

(4) Berendsen, H. J. C.; Grigera, J. R.; Straatsma, T. P. The missing term in effective pair potentials. *J. Phys. Chem.* **1987**, *91*, 6269–6271, DOI: 10.1021/j100308a038.

(5) McQuarrie, D. A. *Statistical Mechanics*; University Science Books: Sausalito, CA, 2000.

(6) Crooks, G. E. Path-ensemble averages in systems driven far from equilibrium. *Phys. Rev. E* **2000**, *61*, 2361–2366, DOI: 10.1103/PhysRevE.61.2361.

(7) Kish, L. *Survey Sampling*; John Wiley & Sons, 1965.

(8) Hardy, G. H.; Littlewood, J. E.; Pólya, G. *Inequalities*; Cambridge: University Press, 1934.

(9) Durkan, C.; Bekasov, A.; Murray, I.; Papamakarios, G. Neural Spline Flows. Advances in Neural Information Processing Systems. 2019; pp 7511–7522.

(10) Bradbury, J.; Frostig, R.; Hawkins, P.; Johnson, M. J.; Katariya, Y.; Leary, C.; Maclaurin, D.; Necula, G.; Paszke, A.; VanderPlas, J.; Wanderman-Milne, S.; Zhang, Q. JAX: composable transformations of Python+NumPy programs. 2018; http://github.com/jax-ml/jax.

(11) Kingma, D. P.; Ba, J. Adam: A Method for Stochastic Optimization. 3rd International Conference on Learning Representations (ICLR). San Diego, CA, USA, 2015.

(12) Frenkel, D.; Smit, B. *Understanding Molecular Simulation: From Algorithms to Applications*, 2nd ed.; Academic Press: San Diego, 2002.
